# On the Rate-Distortion Theory for Task-Specific Semantic Communication

**DOI:** 10.3390/e27080775

**Published:** 2025-07-23

**Authors:** Jingxuan Chai, Huixiang Zhu, Yong Xiao, Guangming Shi, Ping Zhang

**Affiliations:** 1 School of Artificial Intelligence, Xidian University, Xi’an 710126, China; chaijingxuan@stu.xidian.edu.cn (J.C.); gmshi@xidian.edu.cn (G.S.); 2 School of Electronic Information & Communications, Huazhong University of Science & Technology, Wuhan 430074, China; zhuhuixiang@hust.edu.cn; 3 Peng Cheng Laboratory, Shenzhen 518055, China; 4 Pazhou Laboratory (Huangpu), Guangzhou 510335, China; 5 State Key Laboratory of Networking and Switching Technology, Beijing University of Posts and Telecommunications, Beijing 100876, China; pzhang@bupt.edu.cn

**Keywords:** semantic communication, rate-distortion theory, task-specific communication

## Abstract

Semantic communication has attracted considerable interest due to its potential to support emerging human-centric services, such as holographic communications, extended reality (XR), and human-machine interactions. Different from traditional communication systems that focus on minimizing the symbol-level distortion (e.g., bit error rate, signal-to-noise ratio, etc.), semantic communication targets at delivering the intended meaning at the destination user which is often quantified by various statistical divergences, often referred to as the semantic distances. Currently, there still lacks a unified framework to quantify the rate-distortion tradeoff for semantic communication with different task-specific semantic distance measures. To tackle this problem, we propose the task-specific rate-distortion theory for semantic communication where different task-specific statistic divergence metrics can be considered. To investigate the impact of different semantic distance measures on the achievable rate, we consider two popular tasks, classification and signal generation. We present the closed-form expressions of the semantic rate-distortion functions for these two different tasks and compare their performance under various scenarios. Extensive experimental results are presented to verify our theoretical results.

## 1. Introduction

The rapidly growing need for human-oriented communication services has led to the emergence of semantic communication, a novel communication paradigm that focuses on effectively transmitting and delivering the meaning of messages [1,2,3,4,5]. Here, the semantic meaning can be the implicit interpretation of the messages, which may not always be directly observable to users.

Instead of ensuring the bit-level accuracy of the message of delivery, in semantic communication, semantic distance quantifying how well the intended meaning is preserved during transmission is often measured by statistical divergence [2,6,7,8,9]. Generally speaking, different tasks require different divergences to measure their semantic distances because the choice of metrics depends on the nature of the task, the structure of the data, and the desired semantic fidelity. For example, in classification tasks, the Kullback-Leibler (KL) divergence has been commonly considered as the main metrics for measuring the difference between predicted probability distributions and true labels, or for model comparison in Bayesian inference [10]. Also, in generation tasks, the Wasserstein distance is known to exhibit good performance in capturing the geometric relationships between high-dimensional distributions and providing stable gradient flows for generative adversarial networks (GANs) [11].

Despite its promising potential, most existing work focuses on optimizing the semantic communication system based on a single predefined and fixed semantic distance metric [12,13]. There is still lacking a unified information theoretic framework that quantifies the achievable rate for semantic communication under different tasks with different semantic distance measures. In this paper, we propose a novel task-specific rate-distortion theory for semantic communication in which different task-specific statistic divergence-based distance metrics are considered and compared. We consider two popular tasks, classification and signal generation, to investigate the impact of different types of semantic distances on the minimum achievable rate. We present the closed-form expressions of the semantic rate-distortion functions for both tasks and compare their performance under various scenarios. We summarize the main contribution of this paper as follows:

(1) We propose a novel taks-specific semantic communication architecture that generalizes traditional rate-distortion theory by incorporating a general divergence metrics to quantify semantic distance.
(2) We derive the closed-form expressions for the semantic rate-distortion functions under Gaussian semantic sources, specifically for Wasserstein distance, KL divergence, and reverse KL divergence, revealing fundamental tradeoffs among transmission rate, distortion, and semantic distance.
(3) Extensive experiments are conducted on image-based semantic communication systems for both generation and classification tasks. Our results suggest that the proposed framework significantly outperforms traditional MSE-based approaches, with reverse KL divergence demonstrating superior perceptual quality in generation tasks and KL divergence achieving higher classification accuracy.

The rest of this paper is organized as follows: Section 2 introduces the background and preliminaries. The system model and theoretical results are discussed in Section 3 and Section 4, respectively. Section 5 present experimental results and the paper is concluded in Section 6.

## 2. Background and Preliminary

The rate-distortion theory is a branch of information theory that focuses on characterizing the fundamental limit of lossy data compression by the optimal trade-off between the minimum data rate required between the source and the destination (rate) and the maximum tolerable distortion allowed when reconstructing the source data by the destination user based on the received signal (distortion). More formally, for a given distortion level D≥0, the fundamental tradeoff between the minimum achievable rate and maximum tolerable distortion is often represented by the rate-distortion function R(D), defined by

(1) $$\begin{array}{ccc} & & R\left(D\right)=\underset{p_{\hat{S}|S}}{\inf}I\left(S,\hat{S}\right)\\ & & \;\;\;\;\;\;s.t.E[d\left(S,\hat{S}\right)]\leq D, \end{array}$$

where $d(S,\hat{S})$ is the distortion metric that is adopted by the source and destination users for measuring the difference between the original source signal and the signal recovered by the destination. In traditional Shannon rate-distortion theory, *d* is often measured by the symbol-level distortion such as Hamming distortion for discrete signal sources and mean squared error (MSE) distortion for continuous signal sources.

Motivated by the fact that human users are more sensitive to the perceptual quality of the recovered signal, instead of symbol-level recovery, the rate-distortion-perception (RDP) theory has been developed by incorporating the constraint on the statistic divergence of the probability distributions, called perception level, between the source and the recovered signal [12,13]. More formally, for a given maximum-tolerable distortion level *D* and a perception level *P*, the rate-distortion-perception function R(D,P) is defined as

(2) $$\begin{array}{ccc} & & R\left(D,P\right)=\underset{p_{\hat{S}|S}}{\inf}I\left(S,\hat{S}\right)\\ & & \;\;\;\;\;\;s.t.E[d\left(S,\hat{S}\right)]\leq D,\\ & & \;\;\;\;\;\;\;\;\;\;\;\;\theta (p_{S},p_{\hat{S}})\leq P, \end{array}$$

where $\theta (p_{S},p_{\hat{S}})$ is the perception metric that measures the statistical divergence between the source signal distribution and the reconstructed signal distribution. Most existing works on RDP focus on a single perception metrics, such as the total variation (TV) distance [14,15]. Recently, some researchers have investigated the RDP problem under other divergence metrics such as Wasserstein distance [16] and *f*-divergence [17,18,19,20].

In this paper, we investigate the rate-distortion theory of task-specific semantic communication. Our key innovation lies in introducing a generalized divergence metric that quantifies the statistical semantic distance between source and reconstructed signals, where the specific metric is adaptively selected based on communication task requirements. We also obtain the closed-from expressions of the semantic rate-distortion function under various task-specific divergence metrics. A comparison between the existing information-theoretic results of the RDP theory and our results under various types of source signals is shown in Table 1, where the columns of “SI” represent whether or not the corresponding result considers side information.

## 3. System Model and Problem Formulation

### 3.1. System Model

**Figure 1:**
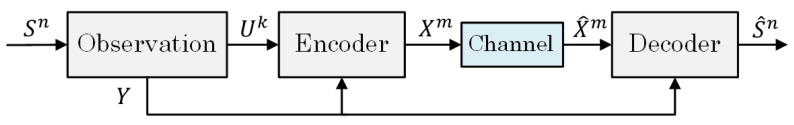
Illustration of the semantic communication model.

We adopt the system model of semantic communication introduced in [2,5], where the primary objective is to effectively convey implicit semantic information to end users by leveraging background knowledge, as depicted in Figure 1. Formally, let the semantic source signal $S^{n}$ represent an *n*-length i.i.d. random variable containing intrinsic information that remains unobservable to the encoder. The encoder accesses only a limited set of *k* indirect observations $U^{k}$ about the semantic source, which may comprise noisy samples or partial observations. The relationship between the source and the observations is characterized by the conditional probability $p_{U|S}$ of obtaining observation *U* given source *S*. Due to the limited channel capacity, the encoder produces an encoded signal sequence $X^{m}$ with its length being *m*. The decoder then receives a potentially corrupted version ${\hat{X}}^{m}$ of this signal through a discrete memoryless channel modeled by $p_{\hat{X}|X}$.

Moreover, we assume that both the encoder and the decoder have access to side information, denoted as *Y*. This may correspond to some background knowledge shared between the source and the destination. For example, in human communication scenarios [5], different users may employ different languages or word sequences to convey the same semantic concept. In such cases, the side information may include the user’s language background and lexical preferences when transforming the semantic information source into observable signals. The main objective of the decoder is to infer the implicit semantic source $\hat{S}$ with a certain level of signal fidelity, based on the signal received from the channel and the available side information.

It can be observed that the above model is similar to the indirect source coding problem with side information in the classic information theory setup. In this paper, we extend the above problem to a more general scenario in which, in addition to MSE distortion, we also consider the impact of task-specific semantic distances on the minimum rate required to recover the implicit signal source. We define the semantic distance as a much broader range of signal fidelity measures, including not only classical distribution divergences such as KL and Wasserstein distance but also the state-of-the-art perceptual quality measures such as the Inception score and SSIM.

We should clarify that the semantic source *S* and its recovery $\hat{S}$ can indeed take traditional forms whether discrete or continuous. Different from traditional communication systems that rely on symbol-level distortion metrics such as Hamming distance and MSE distortion, our proposed semantic communication employs a general task-specific divergence measure to quantify semantic distance. This reflects how effectively the recovered information supports the intended task rather than merely assessing signal-level similarity. Our proposed framework extends classical rate-distortion theory by incorporating the constraint on semantic distance, thereby establishing a theoretical foundation for task-specific communication systems.

Figure 1 illustrates a general semantic communication architecture that encompasses both lossy compression and joint source-channel coding scenarios. In our theoretical analysis, we primarily focus on the lossy compression case with a noiseless channel to clearly demonstrate the fundamental semantic rate-distortion tradeoff.

### 3.2. Problem Formulation

In this paper, we consider the following two types of constraints to measure the fidelity of the signal recovered by the decoder:

(1) *MSE Distortion:* This corresponds to the signal-level distortion measured by the average squared difference of energy between the source and reconstructed signal, defined as $d:S^{n}\times S^{n}\to R$. Denote by D≥0 the maximum MSE distortion level that can be tolerated by the destination user. We can write the following constraint:
  (3) $$C1:\;\;\;E[d\left(S^{n},{\hat{S}}^{n}\right)]\leq D.$$
(2) *Task-relevant Semantic Distance:* This corresponds to the task-specific distribution divergence that measures the semantic dissimilarity between the semantic source and its recovery. Generally speaking, different tasks require different divergence metrics and have different maximum tolerable levels of the recovered signal. For example, KL divergence has been commonly used for signal classification [10,21] and Wasserstein distance has often been adopted for signal generation [11]. In addition to the standard distribution divergence, some novel metrics for measuring the perceptual quality of specific types of signals, such as image and video, including Inception score [22] and SSIM [23], can also be included in our framework. Let M be the set of supported tasks. Let $\theta_{m}$ and $P_{m}$ be the task-specific (semantic) distance metric and the maximum tolerable level for task *m*. We can write the following constraint on the task-relevant semantic distance:
  (4) $$C2:\;\;\;\theta_{m}\left(p_{S^{n}},p_{{\hat{S}}^{n}}\right)\leq P_{m}.$$

We then define a semantic code as follows:

**Definition 1.**  *A semantic code $\left(f_{n},g_{n}\right)$ consists of a encoder, defined as*

(5) $$f_{n}:U^{n}\times Y^{n}\to \left\{1,2,...,2^{nR}\right\},$$

*and a decoder, defined as*

(6) $$g_{n}:\left\{1,2,...,2^{nR}\right\}\times Y^{n}\to {\hat{S}}^{n}.$$

The main objective is to characterize the region of all the achievable tuples $\langle R,D,P_{m}\rangle$ for each specific task. More formally, we first define the notion of achievability as follows:

**Definition 2.**  *A tuple $\langle R,D,P_{m}\rangle$ is achievable if there exists a sequence of codes $\left(f_{n},g_{n}\right)$ that satisfies both constraints C1 and C2.*

Finally, we define the semantic rate-distortion function as follows:

**Definition 3.**  *The task-specific semantic rate-distortion function with side information is defined as the minimum rate required to achieve a signal fidelity that simultaneously satisfies constraints C1 and C2, when the side information Y is available at both the encoder and the decoder, i.e., for task m, we have*

(7) $$R_{m}\left(D,P_{m}\right)=\inf\left\{R:\langle R,D,P_{m}\rangle is\;achievable\right\}.$$

In the rest of this paper, we first present some theoretical results in Section 4 and present experimental results in Section 5. We compare achievable rates across different task requirements through both theoretical derivations and empirical evaluations.

## 4. Theoretical Results

In this section, we first propose the theoretical formulation of the task-specific semantic communication problem to demonstrate how our architecture generalizes generation and classification tasks. We then obtain the closed-form expressions of the Gaussian semantic rate-distortion function under Wasserstein distance, KL divergence and reverse KL divergence.

Since obtaining the exact closed-form solutions of (7) involves a general coding theorem which is out of the scope of this paper, we adopt the following formulation:

(8) $$\begin{array}{ccc} & & R_{m}\left(D,P_{m}\right)=\underset{p_{\hat{S}|UY}}{\inf}I\left(U,\hat{S}|Y\right)\\ & & \;\;\;\;\;\;s.t.E[d\left(S^{n},{\hat{S}}^{n}\right)]\leq D,\\ & & \;\;\;\;\;\;\;\;\;\;\;\;\theta_{m}\left(p_{S^{n}},p_{{\hat{S}}^{n}}\right)\leq P_{m}, \end{array}$$

to characterize the task-specific semantic rate-distortion function. This formulation, which uses conditional mutual information between encoder input and decoder output for the rate term, aligns with established approaches in rate-distortion theory [13,15,16].

It is worth noting that our proposed task-specific semantic communication framework can be extended to a range of applications that can be formulated in terms of statistical divergence minimization, such as Bayesian regression [24] and density estimation [25]. There are, however, many other tasks that require novel signal fidelity measures beyond simple distribution divergence minimization. Novel tasks such as reinforcement learning and clustering, for instance, cannot be adequately formulated using divergence minimization approaches. How these alternative measures on codec design and achievable rates is worth further investigation, which will be left for our future work.

### 4.1. Classification Task

We begin by formalizing our semantic communication framework for the classification task, based on the maximum a posterior probability (MAP) principle [26,27,28].

The classifier outputs the probability of each class label, given by q(U), where q(·) is the coding function of the classifier. We can write the standard classifier based on the MAP principle as follows:

(9) $$\hat{s}=\arg\max_{s\in S}p_{S|U}\left(q(U)|U\right).$$

We can then apply the following divergence with respect to the output of the classification model to develop the classifier. Formally, the loss function under the MAP approach in (1) of [26] can be reformulated as

(10) $$d_{KL}\left(p_{\mathrm{data}},p_{\mathrm{model}}\right)=d_{KL}\left(p_{S},p_{\hat{S}}\right)$$

where $p_{\mathrm{data}}$ is the distribution of true class labels, which is actually the distribution of semantic source $p_{S}$, and $p_{\mathrm{model}}$ is the distribution induced by the classifier, which is actually the distribution of the output probability of the semantic decoder $p_{\hat{S}}=p_{q(U)}$.

Consider the tradeoff relationship between the transmission rate, bit-wise distortion, and the classification accuracy; the objective of the semantic rate-distortion problem in (7) specified for the classification task is defined by:

(11) $$\begin{array}{ccc} & & R_{\mathrm{cls}}\left(D,P_{\mathrm{cls}}\right)=\underset{p_{\hat{S}|UY}}{\inf}I\left(U,\hat{S}|Y\right)\\ & & \;\;\;\;\;\;s.t.E[d\left(S^{n},{\hat{S}}^{n}\right)]\leq D,\\ & & \;\;\;\;\;\;\;\;\;\;\;\;d_{KL}\left(p_{S^{n}},p_{{\hat{S}}^{n}}\right)\leq P_{\mathrm{cls}}, \end{array}$$

where $P_{\mathrm{cls}}$ is the parameter that quantifies the maximum tolerable level of misclassification. The distortion term $E[d\left(S^{n},{\hat{S}}^{n}\right)]$ measures the bit-wise fidelity and the semantic distance term $d_{KL}\left(p_{S^{n}},p_{{\hat{S}}^{n}}\right)$ measures the classification accuracy. This allows us to characterize the tradeoff relationship between the reconstruction quality and the classification accuracy. Note that the mismatch between the random variables in the mutual information term and the constraint terms is a characteristic of indirect source coding problems [29,30]. This formulation extends our general framework while maintaining consistency with the rate-distortion tradeoff established in previous sections. The KL divergence constraint directly links communication efficiency with classification performance through rate-distortion theory.

### 4.2. Generation Task

Our proposed semantic communication architecture can also accommodate the generation task. In this context, the semantic source *S* represents the ground-truth data distribution, while the indirect observation *U* constitutes a noisy or compressed version of *S*. For instance, in image generation scenarios, *S* might correspond to the distribution of high-resolution natural images, while *U* could represent their quantized or downsampled versions. The decoder learns a mapping function that reconstructs an approximation $\hat{S}$ of the original semantic source, where both *S* and $\hat{S}$ share the same alphabet S

Inspired by the existing literature on generative models [11,31], we can adopt Wasserstein distance and reverse KL divergence for exact realizations of the semantic distance that measures the perceptual quality of generative samples. Using the above formulation, the semantic rate-distortion problem in (7) for generation task under Wasserstein distance is then specified by:

(12) $$\begin{array}{ccc} & & R_{\mathrm{gen}}\left(D,P_{\mathrm{gen}}\right)=\underset{p_{\hat{S}|UY}}{\inf}I\left(U,\hat{S}|Y\right)\\ & & \;\;\;\;\;\;s.t.E[d\left(S^{n},{\hat{S}}^{n}\right)]\leq D,\\ & & \;\;\;\;\;\;\;\;\;\;\;\;d_{WD}\left(p_{S^{n}},p_{{\hat{S}}^{n}}\right)\leq P_{\mathrm{gen}}. \end{array}$$

Here, $P_{\mathrm{cls}}$ is the parameter that quantifies the maximum tolerable statistical difference between $p_{S}$ and $p_{\hat{S}}$. Similarly, we can also formulate the semantic rate-distortion problem under reverse KL divergence as follows:

(13) $$\begin{array}{ccc} & & R_{\mathrm{gen}}\left(D,P_{\mathrm{gen}}\right)=\underset{p_{\hat{S}|UY}}{\inf}I\left(U,\hat{S}|Y\right)\\ & & \;\;\;\;\;\;s.t.E[d\left(S^{n},{\hat{S}}^{n}\right)]\leq D,\\ & & \;\;\;\;\;\;\;\;\;\;\;\;d_{RKL}\left(p_{S^{n}},p_{{\hat{S}}^{n}}\right)\leq P_{\mathrm{gen}}. \end{array}$$

The operational significance of the above formulations is demonstrated in Section 5 through the following: their direct implementation as training objectives; empirical validation of the predicted tradeoffs; quantitative comparison to baseline approaches. This formulation allows us to systematically analyze the tradeoffs between compression efficiency *R*, bit-wise reconstruction fidelity *D*, and perceptual quality $P_{\mathrm{gen}}$ in generation-oriented semantic communication systems.

### 4.3. SRD Function for Gaussian Sources

To explore the semantic rate-distortion tradeoff, we derive the closed-form expressions of the semantic rate-distortion functions by considering a particular continuous semantic source, the Gaussian source. More formally, the semantic source is modeled as a standard Gaussian source S∈N(0,1). We assume that the side information also follows a Gaussian distribution, denoted as Y∈N(0,1). The correlation between the semantic source *S* and the side information *Y* is characterized as

(14) $$Y=\eta S+\sqrt{1-\eta^{2}}Z$$

where η∈[0,1] and Z∈N(0,1) is a random variable independent of *S*.

We now present the main results of the Gaussian semantic rate-distortion functions as stated in the following two theorems:

**Theorem 1.**  *The semantic rate-distortion function for Gaussian semantic source N(0,1) under Wasserstein distance is*

(15) $$R_{WD}\left(D,P\right)=\left\{\begin{array}{cc} \frac{1}{2}\log\frac{1-\eta^{2}}{D}, & \mathrm{if}\;0<D<D_{1}^{L}\\ \frac{1}{2}\log\frac{1-\eta^{2}}{1-{\left(\frac{1+\sigma_{1}^{2}-D}{2\sigma_{1}}\right)}^{2}},\; & \mathrm{if}\;D_{1}^{L}\leq D<D_{1}^{R},\\ 0, & \mathrm{if}\;D\geq D_{1}^{R} \end{array}\right.$$

*where $D_{1}^{L}:=\sqrt{P},\;D_{1}^{R}:=1+\sigma_{1}^{2}-2\eta \sigma_{1}$ and $\sigma_{1}=1-\sqrt{P}$.*

**Proof.**  See Appendix A. □

**Theorem 2.**  *The semantic rate-distortion function for Gaussian semantic source N(0,1) under KL divergence is*

(16) $$R_{KL}\left(D,P\right)=\left\{\begin{array}{cc} \frac{1}{2}\log\frac{1-\eta^{2}}{D}, & \mathrm{if}\;0<D<D_{2}^{L}\\ \frac{1}{2}\log\frac{1-\eta^{2}}{1-{\left(\frac{1+\sigma_{2}^{2}-D}{2\sigma_{2}}\right)}^{2}},\; & \mathrm{if}\;D_{2}^{L}\leq D<D_{2}^{R},\\ 0, & \mathrm{if}\;D\geq D_{2}^{R}, \end{array}\right.$$

*and that under reverse KL divergence is*

(17) $$R_{RKL}\left(D,P\right)=\left\{\begin{array}{cc} \frac{1}{2}\log\frac{1-\eta^{2}}{D}, & \mathrm{if}\;0<D<D_{3}^{L}\\ \frac{1}{2}\log\frac{1-\eta^{2}}{1-{\left(\frac{1+\sigma_{3}^{2}-D}{2\sigma_{3}}\right)}^{2}},\; & \mathrm{if}\;D_{3}^{L}\leq D<D_{3}^{R},\\ 0, & \mathrm{if}\;D\geq D_{3}^{R}, \end{array}\right.$$

*where $D_{r}^{L}:=1-\sigma_{2}^{2},D_{2}^{R}:=1+\sigma_{2}^{2}-2\eta \sigma_{2}$, and $D_{3}^{L}:=1-\sigma_{3}^{2},D_{3}^{R}:=1+\sigma_{3}^{2}-2\eta \sigma_{3}$. Here, $\sigma_{2},\sigma_{3}\in \left[0,1\right]$ are the unique solutions to*

(18) $$\begin{array}{cc} \; & \ln\sigma_{2}^{2}+\frac{1}{\sigma_{2}^{2}}-1-2P=0,\\ \; & \ln\sigma_{3}^{2}-\sigma_{3}^{2}+1+2P=0, \end{array}$$

*respectively.*

**Proof.**  See Appendix A. □

We note that, taking P→∞, we have

(19) $$R_{WD}\left(D,\infty \right)=R_{KL}\left(D,\infty \right)=R_{RKL}\left(D,\infty \right)=R\left(D\right)=\frac{1}{2}\log\frac{1-\eta^{2}}{D},$$

where R(D) is the classical Gaussian rate-distortion function with side information (see the Wyner–Ziv theorem in [32]). This indicates that, when the constraint on statistical difference is inactive, the semantic rate-distortion functions under Wasserstein distance, KL divergence, and reverse KL divergence all degenerate into traditional rate-distortion functions. When the side information is independent of the source *S* (i.e., taking η=0), the semantic rate-distortion function $R_{W}\left(D,P\right)$ in Theorem 1 reduces to the rate-distortion-perception function derived in [16]. The above consistency of our results with prior works confirms both the generalization capability and the correctness of our theoretical results.

Based on Theorems 1 and 2, we can quantify the rate difference between the Gaussian semantic rate-distortion function and the traditional rate-distortion function. Formally, taking Wasserstein distance as an example, we have

(20) $$R_{WD}\left(D,P\right)-R\left(D\right)=\frac{1}{2}\log\frac{D}{1-{\left(\frac{1-(P+D)/2}{1-\sqrt{P}}\right)}^{2}}.$$

We can observe that the above rate difference is always positive. Moreover, this rate difference increases with *D* and decreases with *P*. This indicates that, under the same distortion level, the extra rate to satisfy the constraint on semantic distance increases when this constraint becomes tighter. This additional rate requirement decreases when lower bit-wise quality is acceptable.

From Theorem 2, we can derive an inequality relationship between the Gaussian semantic rate-distortion functions under KL divergence and reverse KL divergence:

(21) $$R_{KL}\left(D,P\right)\geq R_{RKL}\left(D,P\right),$$

where the equality holds if and only if P=∞. This means that, under the same *D* and *P*, it generally requires a higher rate to meet the constraint on KL divergence, compared to reverse KL divergence. This also demonstrates that reverse KL divergence is a more rate-efficient metric for preserving the source distribution under the same fidelity requirements. Moreover, the equality condition P=∞ indicates that both divergence metrics converge to the same rate-distortion function when the semantic distance constraint becomes inactive. This aligns with the observation of Theorem 2 that both functions degenerate to the classical Wyner–Ziv rate-distortion function in this limit. The theoretical inequality is further supported by the experimental results in Section 5, where generation tasks using reverse KL divergence achieve better perceptual quality at lower rates.

When $D\leq D_{1}^{L}$ (resp. $D\leq D_{2}^{L}$, $D\leq D_{3}^{L}$), the semantic rate-distortion functions under Wasserstein distance (resp. KL divergence, reverse KL divergence) also coincide with the classical rate-distortion function with side information R(D). This is because, when the distortion constraint is tight, the optimal coding scheme of the classical Shannon rate-distortion problem is able to satisfy the constraint on the distribution-wise semantic distance. Therefore, there is no need to require extra rate to meet the constraints on statistical divergence.

**Figure 2:**
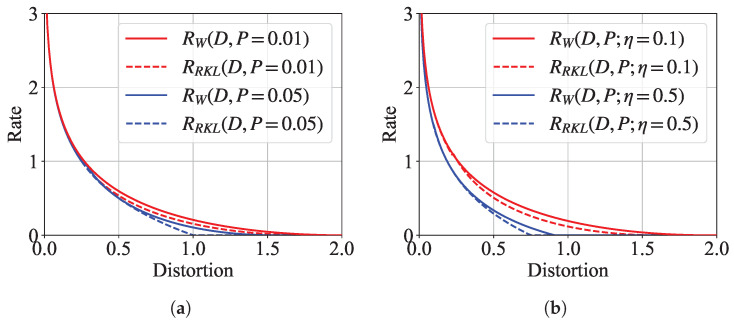
Curve plots of Gaussian semantic rate-distortion functions. (**a**) Rate-distortion curves under some fixed perception level; (**b**) Rate-distortion curves under same perception level and some fixed η values.

In Figure 2a, we plot the curves of semantic rate-distortion functions using the results of Theorems 1 and 2. We observe a three-way tradeoff among transmission rate, bit-wise distortion, and distribution-wise semantic distance. Specifically, given the distortion level, an increase in transmission rate leads to higher semantic fidelity, i.e., smaller semantic distance. Similarly, given the transmission rate, an increase in semantic fidelity leads to lower bit-wise quality, i.e., higher MSE distortion. This observation is aligned with the existing rate-distortion-perception tradeoff [13].

We further observe that, for a given semantic distance level and MSE distortion level, the semantic rate-distortion function under KL divergence consistently exceeds that under the Wasserstein distance. This suggests that KL divergence imposes a stricter measure of statistical difference than the Wasserstein distance for Gaussian sources. However, the semantic rate-distortion function can only capture one aspect of the difference between these two divergence metrics. A more comprehensive discussion is made in the experiment section by investigating the task performance under different divergence metrics for practical sources of real-world datasets.

Figure 2b illustrates the semantic rate-distortion curves under varying side information conditions. The parameter η represents the covariance between *S* and *Y*, serving as a measure of their statistical dependence. A higher η indicates a stronger correlation between the semantic source *S* and the side information *Y*, while η=0 corresponds to complete independence between them.

As shown in Figure 2b, the semantic rate-distortion function varies significantly with η. Specifically, for a given distortion level *D*, the required semantic rate decreases as η increases. Furthermore, the maximum achievable distortion diminishes with larger values of η. These observations suggest that side information effectively acts as shared background knowledge between the encoder and the decoder, facilitating more efficient semantic communication. The stronger the correlation, i.e., the higher η, the greater the reduction in the required rate for a given level of reconstruction fidelity.

## 5. Experimental Results

In this section, we conduct experiments on practical semantic sources and demonstrate the simulation results for our semantic communication system for generation and classification tasks.

### 5.1. Experimental Setups

We consider a semantic communication system in which the semantic information source *S* corresponds to image signals that are uniformly randomly sampled from a given image dataset. The indirect observation *U* is the quantized version of the semantic source. Specifically, the indirect observation process is represented as $U=Q(S;L_{o})$, where $Q(\cdot ;L_{o})$ denotes a uniform quantizer with $L_{o}$ quantization levels. The side information is some intrinsic feature of the semantic source, denoted as $Y=f_{Y}\left(U\right)$, where *Y* denotes the side information of *U* and $f_{Y}$ denotes the function that outputs the feature of *U*, parameterized by deep neural nets (DNNs). The output of the feature extraction net $f_{Y}$ is a low-dimensional feature vector, which is then concatenated with both the encoder and decoder inputs, serving as side information available to the encoder and the decoder. Denote by $d_{Y}$, the dimension of the feature vector of the side information. The encoder maps the indirect observation *u* into a *d*-dimensional latent feature vector, whose entries are then uniformly quantized to $L_{r}$ levels to obtain the output message. The decoder finally recovers the semantic information based on the received message and the side information. The entire process of semantic source coding can be formulated as

(22) $$\hat{S}=f_{D}\left(f_{E}\left(U,Y\right),Y\right),$$

where $f_{E}$ and $f_{D}$ denote the encoding and decoding functions. Note that the uniform quantization of the encoder gives an upper bound of $d\log(L_{r})$ for rate *R*. We measure symbol-based signal distortion using the mean-squared-error (MSE), denoted as $E(∥S-\hat{S}{∥}^{2})$. For a fixed transmission rate, the objective of the semantic rate-distortion function in (12) under Wasserstein distance can be formulated in terms of the following loss function:

(23) $$L_{\mathrm{gen}}=E(||S-\hat{S}{\left|\right|}^{2})+\lambda_{\mathrm{gen}}\cdot d_{WD}\left(p_{S},p_{\hat{S}}\right),$$

where $\lambda_{\mathrm{gen}}$ is a tuning parameter that controls the tradeoff between the bit-wise quality and perceptual quality. Similarly, the loss function for generation under reverse KL divergence is formulated as

(24) $$L_{\mathrm{gen}}=E(||S-\hat{S}{\left|\right|}^{2})+\lambda_{\mathrm{gen}}\cdot d_{RKL}\left(p_{S},p_{\hat{S}}\right),$$

We obtain the experimental SRD curves by evaluating the distortion and perceptual quality of our model on the test set of the image dataset. Different semantic rate-distortion points are obtained by training the model under different desired settings. For the classification task, the objective of the semantic rate-distortion function in (11) for classification can be obtained by minimizing the following loss function:

(25) $$L_{\mathrm{cls}}=E(||S-\hat{S}{\left|\right|}^{2})+\lambda_{\mathrm{cls}}\cdot d_{KL}\left(p_{S},p_{\hat{S}}\right),$$

where $\lambda_{\mathrm{cls}}$ is a tuning parameter that controls the tradeoff between the bit-wise quality and classification performance.

### 5.2. Results for Generation Tasks

In this section, we present the experimental results of generation tasks and investigate the impact of different divergences on perceptual quality. Here, we consider two specific realizations of the divergence function for semantic distance, Wasserstein distance and reverse KL divergence. This choice aligns with our theoretical analysis of Gaussian semantic rate-distortion functions. We also compare our framework with the VAE-based semantic coding paradigm, where a variational codec is optimized for MSE distortion and standard KL divergence.

More specifically, we conduct experiments on two widely used benchmark datasets: MNIST and CIFAR10. The MNIST dataset, consisting of 28 × 28 grayscale handwritten digits, serves as a simple yet effective semantic source to demonstrate the tradeoffs between rate, distortion, and perceptual quality. To further evaluate the scalability of our approach, we also employ the more complex CIFAR10 dataset, which contains 32 × 32 color images across 10 object categories. For both datasets, the semantic source *S* corresponds to the original images, while the indirect observation *U* is their quantized version.

#### 5.2.1. Achievable Rates Under Different Distance Measures

**Figure 3:**
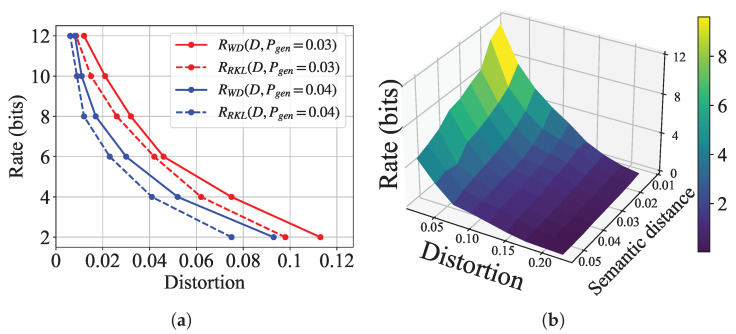
Experimental semantic rate-distortion functions for generation task. (**a**) Curve plots of semantic rate-distortion functions under different divergence metrics; (**b**) Surf plot of 3D semantic rate-distortion function.

Figure 3a illustrates the simulation semantic rate-distortion curves under Wasserstein distance and reverse KL divergence, denoted by $R_{WD}$ and $R_{RKL}$, respectively. We observe a three-way trade-off among transmission rate, MSE distortion, and semantic distance: For a fixed distortion level, increasing the transmission rate leads to smaller semantic distance. Conversely, under a fixed rate, stricter semantic distance constraints result in higher bit-wise distortion. To obtain a more intuitive understanding of the semantic rate-distortion tradeoff, we also demonstrate a 3D surf plot of the semantic rate-distortion function in Figure 3b.

Moreover, we also note that the rate under Wasserstein distance to achieve the same levels of distortion and semantic distance is always higher than that under reverse KL divergence. This confirms that Wasserstein distance imposes a more stringent measure of statistical dissimilarity, as noted in Section 4.3. The above observations are aligned with the theoretical predictions for Gaussian sources in Theorems 1 and 2.

#### 5.2.2. Impact of Indirect Observation

**Figure 4:**
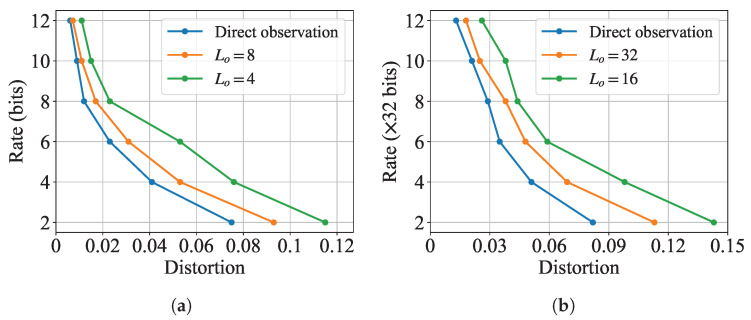
Experimental semantic rate-distortion functions for generation task under different $L_{o}$ values. (**a**) Results on MNIST; (**b**) Results on CIFAR10.

To investigate the impact of the indirect observation process, we demonstrate the simulation semantic rate-distortion function under different settings of the quantization levels $L_{o}$ of the indirect observation in Figure 4. Here, “direct observation” corresponds to the case where the encoder directly observes the original samples from semantic source *S*. From the simulation results on MNIST in Figure 4a and those on CIFAR10 in Figure 4b, we observe that the required rate to achieve the same distortion level increases when the quantization level decreases. This phenomenon can be attributed to the information loss incurred during the quantization process, which forces the system to compensate through higher channel capacity.

Moreover, our experiments reveal a notable difference in how indirect observation affects performance across the MNIST and CIFAR10 datasets. While both datasets exhibit degraded performance under coarser quantization, the impact is significantly more pronounced for CIFAR10. Specifically, for MNIST at 12 bits, direct observation achieves MSE = 0.0061, while quantization degrades performance to 0.0072 ($L_{o}=8,+18\%$) and 0.011 ($L_{o}=4,+80\%$). For CIFAR10 at equivalent 384 bits (12 × 32), MSE increases from 0.013 (direct) to 0.018 ($L_{o}=32,+38\%$) and 0.026 ($L_{o}=16,+100\%$). This discrepancy stems from the inherent complexity difference between the two datasets. The CIFAR10 dataset contains more complex visual features and higher-dimensional structures, rendering it more susceptible to information degradation during indirect observation. In contrast, the MNIST dataset’s simpler digit patterns and limited variability demonstrate greater resilience to quantization artifacts. This observation suggests that semantic communication systems handling complex, real-world data require more careful design of the observation process compared to those processing simpler, structured data.

#### 5.2.3. Impact of Side Information

**Figure 5:**
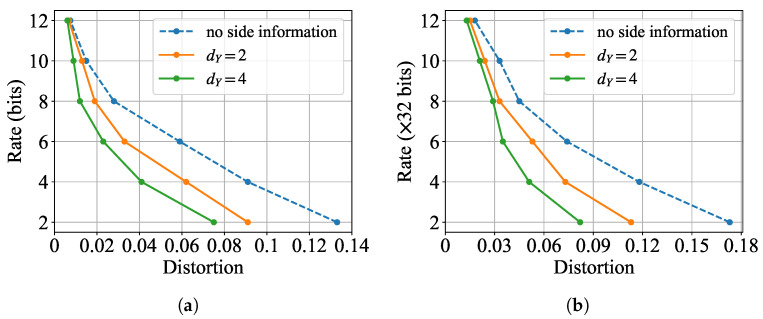
Experimental semantic rate-distortion functions for generation task under different values of $d_{Y}$. (**a**) Results on MNIST; (**b**) Results on CIFAR10.

In order to observe the role of side information in the proposed semantic communication systems, we also conduct experiments under different settings of side information. Figure 5 demonstrates the simulation semantic rate-distortion curves under different settings of the dimensions of feature vectors of side information, on both MNIST and CIFAR10 datasets. Both the results in Figure 5a and those in Figure 5b show that the required rate can be reduced when side information at the encoder and the decoder is accessible, and the reduced rate increases with the the amount of information in *Y*, as measured by the value of $d_{Y}$. The above observations are aligned with the theoretical results of the Gaussian semantic rate-distortion functions in Theorems 1 and 2. This again justifies the fact that offering side information as the background knowledge of the users can effectively save communication resources.

Moreover, we observe that the reduced rate due to the existence of side information is much higher at low rates than at high rates, demonstrating that side information plays a more crucial role when channel capacity is limited. For the MNIST dataset, at the lowest rate of 2 bits, the MSE distortion decreases significantly from 0.133 (no side information) to 0.091 ($d_{Y}=2,-31.6\%$) and further to 0.075 ($d_{Y}=4,-43.6\%$). In contrast, at the highest rate of 12 bits, the distortion only reduces from 0.0075 to 0.0068 (−9.3%) and 0.0061 (−18.7%) for $d_{Y}=2$ and $d_{Y}=4$, respectively. A similar trend is observed for CIFAR10. At 64 bits, the distortion drops substantially from 0.173 to 0.113 (−34.7%) and 0.082 (−52.6%) when side information is introduced, while at 384 bits (12 × 32) the improvement is more modest, from 0.018 to 0.015 (−16.7%) and 0.013 (−27.8%). These results clearly indicate that side information provides the greatest relative benefit in low-rate regimes where the channel capacity is severely constrained. The additional background knowledge helps compensate for the lack of transmission bandwidth, enabling more efficient semantic communication. As the rate increases, the marginal utility of side information decreases, as the system can rely more on the primary channel to convey necessary information. This observation aligns with theoretical expectations and reveals the importance of incorporating side information, particularly in bandwidth-limited scenarios.

#### 5.2.4. Perceptual Quality Under Different Divergences

**Figure 6:**
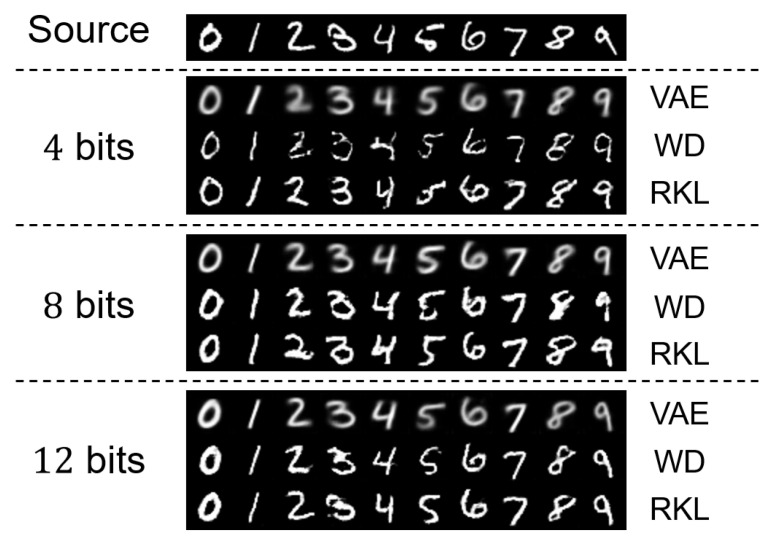
Visual results of the generated image samples under different divergences. The “Source” row displays the ground-truth images available at the encoder. The subsequent rows show reconstructed images at transmission rates of 4 bits, 8 bits, and 12 bits. The columns labeled “WD” and “VAE” demonstrate reconstruction results using our proposed semantic communication architecture optimized for Wasserstein distance and reverse KL divergence respectively.

In Figure 6, we demonstrate the visual results of generated image samples of our proposed model under Wasserstein distance and reverse KL divergence. We also consider a baseline model where a VAE encoder–decoder pair is adopted for our framework. We see that a higher transmission rate results in increased perceptual quality, which coincides with the theoretical observation of the tradeoff relationship between rate and semantic distance for Gaussian semantic rate-distortion functions.

We also observe that the generative quality of our proposed models consistently outperforms the traditional VAE-based coding scheme. The method optimizing reverse KL divergence produces images with the best perceptual quality. This is aligned with the empirical observation in [31] that leveraging reverse KL divergence can improve the sharpness of generated samples. The inferior perceptual quality of VAE-based approaches can be attributed to their optimization objective: by minimizing standard KL divergence, VAEs prioritize generating easily classifiable samples at the expense of human-oriented perceptual fidelity [31]. This fundamental tradeoff between discriminative performance and perceptual quality underscores the advantages of our proposed divergence metrics for semantic communication.

In order to make a fair comparison between the perceptual quality of generated samples under different divergence constraints, we adopt a widely used metric that quantifies the perceptual quality of generated images, the Inception score (IS) [22]. It provides a way of measuring both the clarity of the generated images and their diversity. Formally, the IS of an image source $p_{G}$ is calculated using the following formula:

(26) $$\mathrm{IS}=\exp\left(H_{p}\left(K\right)-E_{X∼p_{G}}\left[H_{p_{G}}\left(K|\hat{S}\right)\right]\right)$$

where *p* is the induced distribution of the Inception-V3 network, $\hat{S}$ is the random variable of generated images, and *K* is the corresponding class label of $\hat{S}$. The generative quality of generated images increases with IS.

**Figure 7:**
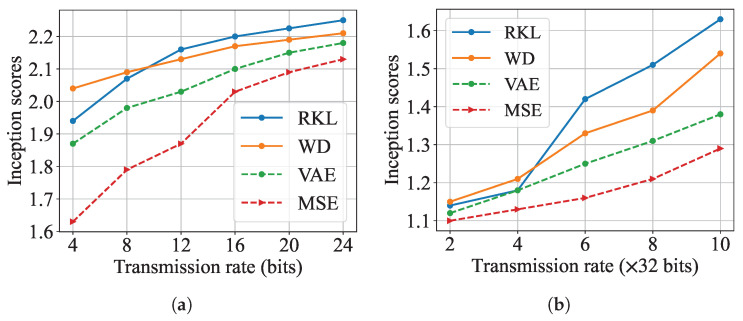
Image quality evaluation of generated samples on MNIST under different divergence metrics. (**a**) Results on MNIST; (**b**) Results on CIFAR10.

In Figure 7, we present the comparison of Inception scores across the evaluated models at varying transmission rates. Here, “MSE" denotes the model minimizing MSE distortion only, which is realized by taking $\lambda_{\mathrm{gen}}=0$ in the loss function in (23). Both the results on MNIST in Figure 7a and those on CIFAR10 in Figure 7b demonstrate clear positive correlation between transmission rate and Inception score for all models, which directly corroborate the rate-semantic distance trade-off relationship illustrated in Figure 4a. This observation confirms our theoretical expectation that higher transmission rates enable better preservation of semantic information, as reflected in improved Inception scores. Moreover, we observe that the models with additional divergence constraints produce images with higher perceptual quality with a huge margin, particularly at low-bit regimes, compared to the model that implicitly minimizes the MSE distortion. This means that our proposed architecture is able to obtain much higher human-oriented perceptual quality compared to traditional bit-wise loss function-based models.

Furthermore, the relative performance ranking among models, with KL and WD consistently outperforming the VAE baseline, aligns precisely with the visual comparisons shown in Figure 6. This consistency between quantitative metrics and human perceptual evaluation strengthens the validity of our experimental findings and provides comprehensive evidence for the superiority of our proposed approaches.

### 5.3. Results for Classification Tasks

To further evaluate the effectiveness of our proposed semantic communication framework across different tasks, we conduct classification experiments using the MNIST dataset under different divergence metrics.

**Figure 8:**
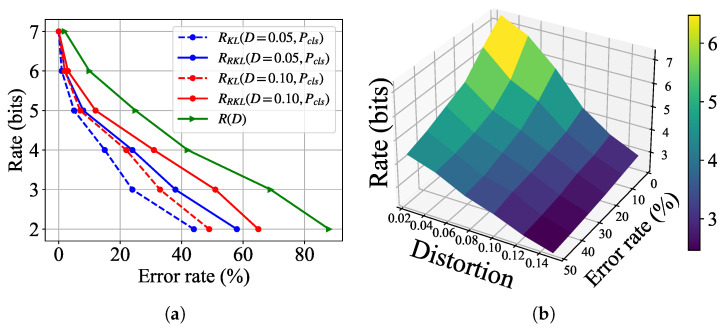
Experimental semantic rate-distortion functions for classification tasks on MNIST dataset. (**a**) Rate-distortion curves; (**b**) Three-dimensional surface plot.

In Figure 8a, we present the classification accuracy of the recovered images under KL divergence and reverse KL divergence. Here, R(D) represents the simulation curves under only MSE distortion, which is obtained by setting $\lambda_{\mathrm{cls}}=0$ in (25). The results demonstrate that, at any given transmission rate, our proposed models achieve significantly higher classification accuracy compared to conventional bit-wise approaches, highlighting the effectiveness of our task-specific semantic communication architecture. Moreover, the results exhibit the following tradeoff relationship: (1) higher transmission rates consistently lead to improved classification accuracy across all models; (2) a more stringent constraint on semantic distance, i.e., higher $P_{\mathrm{gen}}$, results in higher classification accuracy. This also aligns with the theoretical and empirical findings of the semantic rate-distortion tradeoff, confirming that increased transmission rates enhance the preservation of semantic information. A 3D surf plot of the semantic rate-distortion function for the classification task is shown in Figure 8b.

Our experiments also reveal a significant divergence in task performance between the KL-optimized and reverse KL-optimized models. As shown in Figure 8a, the standard KL divergence approach demonstrates superior classification accuracy compared to its reverse KL counterpart, particularly in low-rate regimes (R≤5 bits) where the performance gap exceeds 11% when R= bits and P=0.01. This empirical observation corroborates fundamental information-theoretic principles: the forward KL divergence directly minimizes the expected negative log-likelihood of the true distribution under the approximated distribution [33], making it theoretically optimal for discriminative tasks like classification.

The performance advantage diminishes at higher transmission rates (R>6 bits), where both divergence metrics achieve comparable accuracy within a 2% margin. This convergence suggests that sufficient channel capacity can compensate for the inherent limitations of reverse KL divergence in classification scenarios. The model minimizing KL divergence to produce distributions with “mode-seeking" behavior [34], while beneficial for perceptual quality, as shown in Figure 6 and Figure 7a, appears less suitable for preserving the complete feature distributions needed for robust classification at low bitrates.

In summary, the classification results validate the practical utility of our semantic communication framework to classification tasks, demonstrating the flexibility of the proposed architecture to different tasks.

## 6. Conclusions

In this paper, we have investigated the fundamental limits of task-specific semantic communication by extending the traditional rate-distortion theory to incorporate semantic distance constraints, as measured by a general divergence metric. We have considered a semantic communication model where the encoder accesses the semantic information source only through indirect observations, while both the encoder and the decoder utilize available side information. Our framework specifically addresses classification and generation tasks in task-specific semantic communication systems. We have derived the closed-form expressions for Gaussian semantic rate-distortion functions under various divergence metrics, revealing a fundamental three-way tradeoff involving transmission rate, bit-wise distortion, and distribution-wise semantic distance. Experimental validation using image-based semantic sources confirms these theoretical findings.

## Figures and Tables

**Table 1:** A summary of information-theoretic results in RDP theory. For Bernoulli case, the source follows a Bernoulli distribution with parameter π. For Gaussian case, the source follows a Gaussian distribution N(0,1). Here $D_{1}=\frac{P}{1-2\pi -2P}$, $D_{2}=2\pi \left(1-\pi \right)-\left(1-2\pi \right)P$.

Divergence	Source	R(D,P)	SI	Work
TV	Bernoulli	$\left\{\begin{array}{cc} H_{b}\left(\pi \right)-H_{b}\left(D\right), & \;D\in [0,D_{1})\\ 2H_{b}\left(\pi \right)+H_{b}\left(\pi -P\right)-H_{t}\left(\frac{D-P}{2},\pi \right)\\ -H_{t}\left(\frac{D+P}{2},1-\pi \right), & \;D\in [D_{1},D_{2})\\ 0, & \;D\in [D_{2},\infty ] \end{array}\right.$	✗	[13]
WD	Gaussian	$\left\{\begin{array}{cc} \frac{1}{2}\log\frac{1}{1-{\left(\frac{{\left(1-\sqrt{P}\right)}^{2}+1-D}{2-2\sqrt{P}}\right)}^{2}}, & \sqrt{P}\leq 1-\sqrt{\left|1-D\right|}\\ \max\{\frac{1}{2}\log\frac{1}{D},0\}, & \sqrt{P}>1-\sqrt{\left|1-D\right|} \end{array}\right.$	✗	[16]
WD, KL, RKL	Gaussian	Equations (15)–(17)	✔	Proposed

## Data Availability

The MNIST and CIFAR10 datasets used in this study are publicly available through the PyTorch library. The datasets can be accessed via the PyTorch official documentation: https://pytorch.org/vision/stable/datasets.html.

## Appendix A. Proof of Theorems 1 and 2

To begin with, we note that the mutual information between two Gaussian distributions is independent of their means. Therefore, without loss of generality, we may assume that the recovery source $\hat{S}$ and the semantic source *S* share the same mean. Specifically, we let $\hat{S}∼N\left(0,\sigma^{2}\right)$ for analytical convenience.

We first discuss the case where the constraint on Wasserstein distance is not active, i.e.,

(A1) $$d_{WD}\left(p_{S},p_{\hat{S}}\right)={\left(1-\sigma \right)}^{2}<P.$$

In this case, based on the Wyner–Ziv theorem, we have

(A2) $$\begin{array}{c} R_{\alpha }\left(D,P\right)=\frac{1}{2}\log\frac{1-\eta^{2}}{D}, \end{array}$$

with

(A3) σ=1−D.

Taking (A3) back into (A1), we have

(A4) $$0\leq D<\sqrt{P}=D_{1}^{L}.$$

When the constraint on Wasserstein distance is active, i.e.,

(A5) $$d_{WD}\left(p_{S},p_{\hat{S}}\right)={\left(1-\sigma \right)}^{2}=P,$$

we have

(A6) $$\sigma =1-\sqrt{P}=\sigma_{1}.$$

Then, define the following coding schemes

(A7) $$\left(Y,S,\hat{S}\right)=\left(\eta S^{\prime }+\sqrt{1-\eta^{2}}Y^{\prime },S^{\prime },bS^{\prime }+\sqrt{1-b^{2}}{\hat{S}}^{\prime }\right),$$

where $\left(Y^{\prime },S^{\prime },{\hat{S}}^{\prime }\right)$ is standard normal and

(A8) $$b=\sqrt{\frac{\kappa^{2}-\eta^{2}}{1+\eta^{2}\kappa^{2}-2\eta^{2}}},$$

which gives us $E\left[E{\left[S|\hat{S},Y\right]}^{2}\right]=\kappa^{2}$. Let $\hat{S}=\sigma_{1}\kappa^{-1}E\left[S|\hat{S},Y\right]$ with $\kappa =\left(1+\sigma_{1}^{2}-D\right)/2\sigma_{1}$, we then have

(A9) $$\begin{array}{cc} \; & E\left[d\left(S,\hat{S}\right)\right]=E\left[{\left(S-\kappa^{-1}E\left[S|\hat{S},Y\right]\right)}^{2}\right]\\ = & E\left[S^{2}\right]+\left(\sigma_{1}^{2}\kappa^{-2}-2\sigma_{1}\kappa^{-1}\right)E\left[E{\left[S|\hat{S},Y\right]}^{2}\right]\\ = & 1+\sigma_{1}^{2}-2\sigma_{1}\kappa =D, \end{array}$$

which is equivalent to $E[d\left(S,\hat{S}\right)]=D$. The mutual information term is then written as

(A10) $$I\left(S;\hat{S}|Y\right)=h\left(S|Y\right)-h\left(S|\hat{S},Y\right)=\frac{1}{2}\log\frac{1-\eta^{2}}{1-\kappa^{2}}.$$

The mutual information $I(S;\hat{S}|Y)$ equals zero when κ=η, i.e.,

(A11) $$\begin{array}{cc} \; & \left(1+\sigma_{1}^{2}-D\right)/2\sigma_{1}=\eta \\ \Leftrightarrow & D=1+\sigma_{1}^{2}-2\eta \sigma_{1}\; \end{array}$$

This concludes the proof of Theorem 1. By replacing the constraint on Wasserstein distance with the following KL divergence

(A12) $$d_{KL}\left(p_{S},p_{\hat{S}}\right)=\frac{1}{2}\left(\ln\sigma^{2}+\frac{1}{\sigma^{2}}-1\right)\leq P,$$

and reverse KL divergence

(A13) $$d_{RKL}\left(p_{S},p_{\hat{S}}\right)=\frac{1}{2}\left(-\ln\sigma^{2}+\sigma^{2}-1\right)\leq P,$$

and repeating the same steps of the proof of Theorem 1, we conclude the proof of Theorem 2.
